# A Curious Case of Chryseobacterium indologenes Culture in a Young Adult Kidney Transplant Patient

**DOI:** 10.7759/cureus.33395

**Published:** 2023-01-05

**Authors:** Riddhi R Machchhar, Jessica S Yang, Marciano Figueroa, Kajal Ghodasara, Bassam Hasan

**Affiliations:** 1 Internal Medicine, Hackensack Meridian Health Ocean University Medical Center, Brick, USA; 2 Internal Medicine, Rowan University School of Osteopathic Medicine, Stratford, USA; 3 Infectious Diseases, Hackensack Meridian Health Ocean University Medical Center, Brick, USA

**Keywords:** immunocompromised patient, trimethoprim/sulfamethoxazole, multi-drug resistant organism (mdro), symptomatic uti, chryseobacterium indologenes

## Abstract

*Chryseobacterium indologenes* (*C. indologenes*) is an increasingly common multidrug-resistant organism (MDRO) and is not part of the normal human flora. It is most commonly found in patients who are immunocompromised and/or in poor health, with multiple comorbidities. As an increasingly identified MDRO, *C. indologenes* needs to be identified early, especially in patients with multiple comorbidities, organ transplants, or on mechanical ventilation. We present a case of a young immunocompromised male with an extensive kidney disease history who acquired this new MDRO bacteria, *C. indologenes*.

## Introduction

*Chryseobacterium indologenes* (*C. indologenes*) is a gram-negative, non-fermenting, non-motile, catalase, oxidase, and indole-positive aerobic bacilli uncommon in humans [1-3]. *C. indologenes* is found in soil, water, plants, and food products. Wet surfaces, like water systems, act as a reservoir, and chlorination cannot eradicate the bacteria. In the early 1990s, this bacterium was first documented in a case of ventilator-associated pneumonia and is identified in immunocompromised and comorbid patients [3]. These patients with low immune systems usually acquire this low-virulence bacteria in the hospital. They can present with bacteremia, peritonitis, pneumonia, empyema, pyelonephritis, cystitis, meningitis, and central venous catheter-associated infections [2]. Indwelling devices like catheters or mechanical ventilation can also increase the chance of contracting this microbe [2,3]. *C. indologenes* grows poorly on MacConkey agar but does well on blood agar after 24 h incubation at 37°C [3]. This bacterium is a rapidly evolving and multidrug-resistant organism (MDRO); thus, blood and urine cultures and obtaining antibiotic susceptibility are important [2,3]. Furthermore, when prescribing antibiotics to patients with comorbidities or compromised organs, considerations should be taken. We report a case of a young adult male with a history of congenital kidney disease and kidney transplant who presented with fevers and periumbilical as well as epigastric pain.

## Case presentation

Our patient is a 26-year-old male who presents to the emergency department (ED) for evaluation due to abdominal pain, periumbilical and epigastric, and a fever of 100.5 F at home. The patient has a history of end-stage renal disease (ESRD) due to congenital kidney disease in which he was born with one aplastic kidney. He was briefly on dialysis and received a deceased donor kidney transplant nine years ago. His baseline creatinine runs 1.2-1.3, and he regularly sees his nephrologist. His last episode of rejection was five years ago. He is compliant with his medications and is currently immunosuppressed from tacrolimus. Our patient has been on tacrolimus and mycophenolate immunosuppressants at the time of his kidney transplant due to a congenital renal disease leading to renal failure. He also intermittently self-catheterizes due to neurogenic bladder and is on cephalexin prophylactically for urinary tract infections. The night before his presentation, he developed a fever and right lower quadrant pain. The following morning the pain was worse, along with persistent fever, so he came to the emergency department to be evaluated. No associated headache, sore throat, coughing, nausea, vomiting, diarrhea, or urinary symptoms exist. The family provided insight at the time of the presentation.

**Figure 1:**
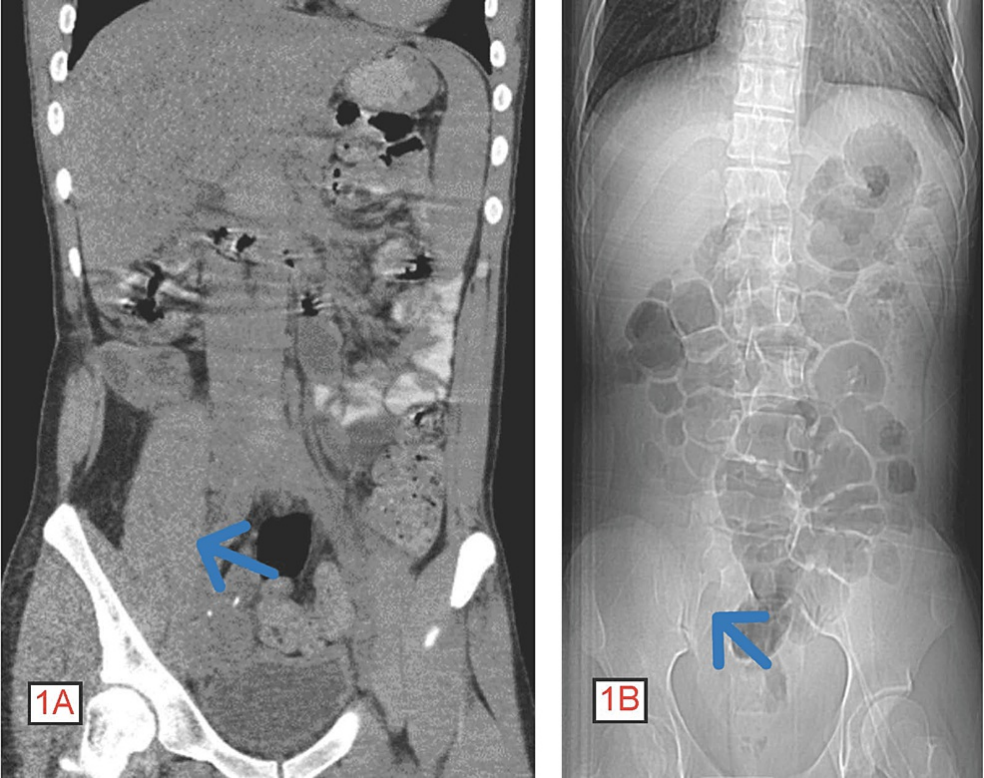
Computed tomography (CT) of the abdomen without contrast depicting no bowel obstruction, an unidentifiable appendix, and no inflammatory changes in the right lower quadrant

In the ED, his maximum temperature was 100.5 F (38.06 C), which was resolved with one dose of acetaminophen. Piperacillin-tazobactam and vancomycin were prescribed, as well. The CT abdomen was negative for hydronephrosis and appendicitis (Figures 1A, 1B).

**Figure 2:**
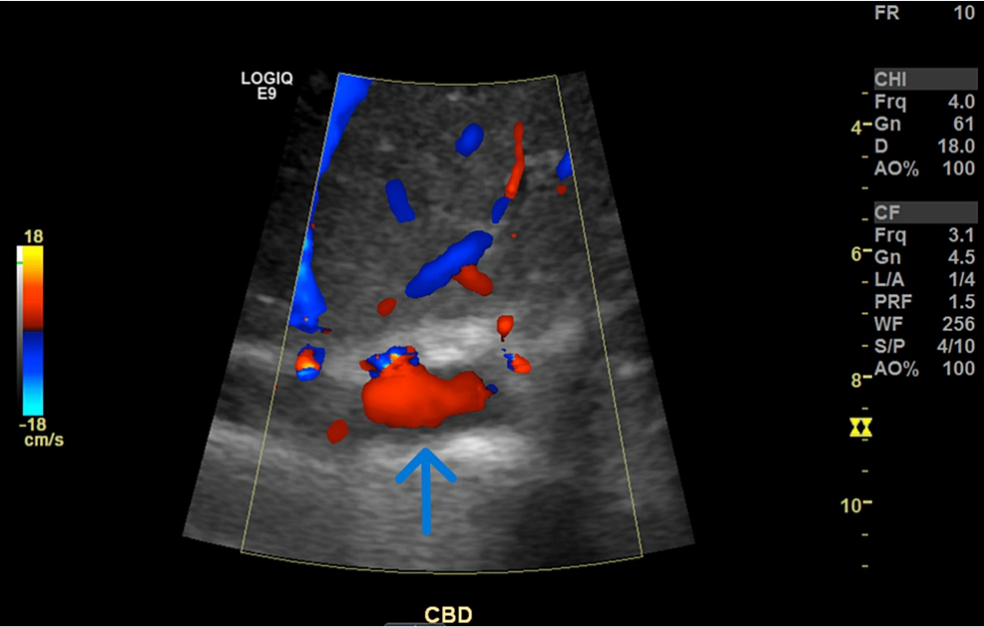
Abdominal ultrasound (US) revealing no intrahepatic ductal dilatation with a nondilated common bile duct (CBD) measuring 3 mm

**Figure 3:**
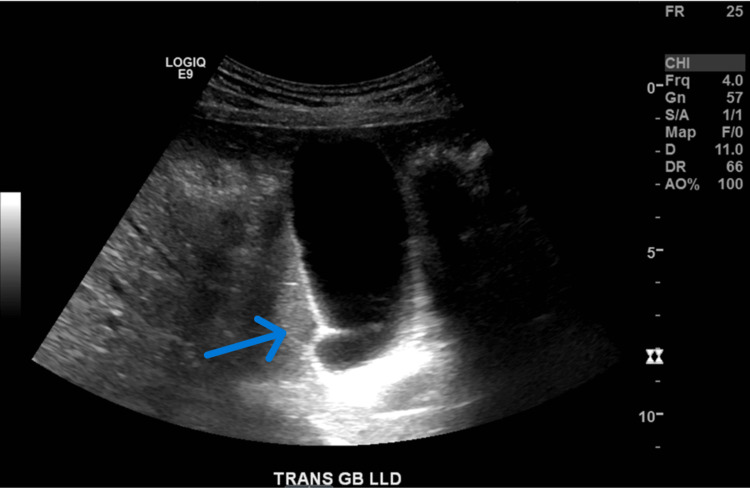
Abdominal ultrasound (US) revealing no stones and minimal gallbladder sludge

He then had an abdominal ultrasound, which revealed an extrarenal pelvis but no hydronephrosis (Figure 2, Figure 3).

**Figure 4:**
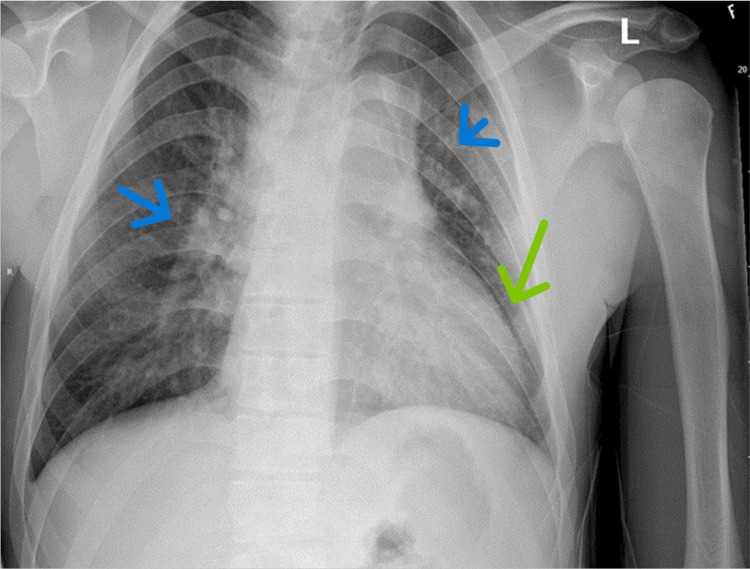
Chest radiograph (CXR) revealing an enlarged cardiomediastinal silhouette (green arrow), and well-inflated lung fields with faint airspace consolidations in the bilateral hilar regions (blue arrows). No pneumothorax was appreciated

His chest X-ray showed faint airspace consolidations in the bilateral hilar regions (Figure 4).

An ultrasound of the renal transplant was unremarkable. The patient was COVID-19 while respiratory syncytial virus (RSV), influenza A, and influenza B polymerase chain reaction (PCR) tested negative. He was admitted for further evaluation. During admission, the patient noted pain in his suprapubic region instead of his right upper quadrant, which prompted a bladder scan. His bladder scan indicated a need to intermittently catheterize the patient if more than 400 mL of urine remained after voiding to alleviate pain.

The patient was also tested for cytomegalovirus in whole blood by real-time PCR and was negative. No Epstein-Barr virus was detected by PCR test.

His urine cultures showed multidrug-resistant organisms (MDRO), proteinuria, WBCs, RBCs, and trace leukocyte esterases. Of significance was a urine culture reflecting 10,000-25,000 CFU/mL *Chryseobacterium indologenes*. Blood cultures were drawn, which showed* Chryseobacterium indologenes*. The lab used matrix-assisted laser desorption/ionization-time of flight (MALDI-TOF) for identification. Trimethoprim/sulfamethoxazole became the antibiotic of choice over piperacillin-tazobactam and vancomycin. His tacrolimus levels returned at 27.1, leading to a hold of his evening dose. Soon after, the tacrolimus levels were regulated, and all further doses resumed. Nephrology followed the patient for acute kidney injury (AKI) and his history of renal transplant and ESRD on tacrolimus (Table 1).

**Table 1:** Laboratory investigations

Clinical Parameters	Day 4	Day 3	Day 2	Day 1
COMPLETE BLOOD COUNT (CBC)				
White Blood Cells	5.1	6.1	6.3	7.2
Red Blood Cells	3.37	2.99	3.27	3.26
Hemoglobin (12.1 to 15.1 g/dL)	10.3	9.3	10.3	10.1
Hematocrit	31.9	27.5	30.1	30.9
Mean Corpuscular Volume	94.7	92.0	92.0	94.8
Platelet Count (1.35-3.17 x 10*6/uL)	168	138	155	135
Neutrophils, percent (40-60%)	65.6	74.5	77.2	72.5
Lymphocytes, percent	19.3	11.1	9.9	15.1
Neutrophils, absolute	3.3	4.5	4.9	5.2
Lymphocytes, absolute	1.0	07	0.6	1.1
CHEMISTRY ROUTINE (CMP)				
Glucose (<140 mg/dL)	100	82	97	94
Blood Urea Nitrogen (6-24 mg/dL)	17	12	15	24
Creatinine (0.6-1.1 mg/dL)	1.15	1.14	1.24	1.38
Sodium (135-145 meq/L)	136	140	139	136
Potassium (3.6-5.2 mmol/L)	4.3	3.3	3.9	4.5
Chloride	101	109	103	105
Anion Gap	8.9	7.0	10.0	4.0
Calcium (8.5-10.2 mg/dL)	9.3	8.0	9.1	8.9
Carbon Dioxide	27	24	26	27
Phosphorus	3.3	3.4	3.7	3.7
Magnesium	1.7	1.2	1.6	2.0
Alkaline Phosphatase (44 - 147 U/L)	74	62	77	80
Protein Total	7.0	5.9	6.6	6.6
Albumin (3.4 - 5.4 g/dL)	4.1	3.5	4.1	4.1
AG Ratio	1.4	1.5	1.6	2.0
Bilirubin, Total (1.2 mg/dL)	0.5	1.1	0.8	0.6
AST (10 - 36 U/L)	16	12	17	16
Iron				31
TIBC				277
Transferrin				198.1
Urinalysis/Microbiology (UA/MICRO)				
Appearance, Urine				Clear
Color, Urine				Yellow
pH, Urine				7.5
Specific Gravity, Urine				1.020
Glucose, Urine				Negative
Blood, UA				Trace
Ketones, UA				Negative
Protein				100.0
Urobilinogen, Urine				0.2
Bilirubin, Urine				Negative
Nitrites, UA				Negative
WBC, UA				3-5
Leukocyte Esterase				Trace
RBC, UA				8-10
Bacteria, UA				Rare

Closer to discharge, the patient was improving; he tolerated his diet and had a physical exam within normal limits. He was discharged home in stable condition with the remaining course of trimethoprim/sulfamethoxazole for another week.

## Discussion

While *Chryseobacterium indologenes* is mainly found in soil and water, it can cause significant morbidity and mortality once colonized in patients who are immunocompromised, and/or severely sick, and/or with in-dwelling devices [4]. It is most commonly found in patients as a nosocomial infection. It is important to note that the bacterium resists chlorination and can be found in municipal water supplies. Notably, there can be *Chryseobacterium indologenes *infections in immunocompetent individuals, including infants, as well [5,6].

The common clinical presentations of *Chryseobacterium indologenes* include pneumonia, bacteremia, cellulitis, surgical wound infections, urinary tract infections, ocular infections, meningitis, peritonitis, intra-abdominal, as well as other shunt and catheter-related infections [2,7-11]. In our case, the patient was immunosuppressed due to his kidney transplant and presented with a UTI due to* Chryseobacterium indologenes*.

It is important to consider* Chryseobacterium indologenes* since it is an emerging organism with changing multidrug resistance. While there was a case of* C. indologenes* successfully treated in a 19-year-old patient presenting with UTI with piperacillin/tazobactam [12], in our patient, the* Chryseobacterium indologenes* was piperacillin/tazobactam resistant and was instead treated with trimethoprim/sulfamethoxazole, as suggested by the susceptibility report, which was determined by CLSI MIC (Table 2).

**Table 2:** Results of MDRO (Chryseobacterium indologenes), antibiotic sensitivity MDRO: multidrug-resistant organism; TMP/SMX: trimethoprim/sulfamethoxazole

Susceptibility	Chryseobacterium indologenes	
Ciprofloxacin	1 ug/mL	Sensitive
Cefazolin	>=64 ug/mL	Resistant
Gentamicin	>=16.0 ug/mL	Resistant
Imipenem	>=16 ug/mL	Resistant
Tobramycin	>=16.0 ug/mL	Resistant
Ceftazidime	>=64 ug/mL	Resistant
Cefepime	>=64 ug/mL	Resistant
Ceftriaxone	>=64 ug/mL	Resistant
TMP/SMX	<=20 ug/mL	Sensitive
Piperacillin/Tazobactam	>=128 ug/mL	Resistant

Due to this, it is suggested to get the local sensitivity pattern as the susceptibility may differ from reports. In a Taiwanese study looking at 84 *C. indologenes *isolates collected from 2005-2017, it was found that the isolates were most susceptible to minocycline (73%), followed by trimethoprim-sulfamethoxazole (47.6%), tigecycline (34.1%), and levofloxacin (32.5%) [13]. Contrastingly, the SENTRY antimicrobial surveillance data with 44 *C. indologenes* isolates (2013-2021) showed the most susceptibility to trimethoprim-sulfamethoxazole (95.5%), piperacillin-tazobactam (86.4%), ceftazidime (68.2%), and cefepime (43.2%). Four* C. indologenes* strains in the SENTRY data showed 75.0% susceptibility to cefoperazone. Resistance patterns in the 44 *C. indologenes* SENTRY strains were the most resistant for colistin (100%), aztreonam (100%), tobramycin (100%), meropenem (93.2%), imipenem (88.6%), gentamicin (84.1%), cefepime (56.8%), and amikacin (47.7%).

In addition, it is necessary to consider risk factors in patients, whether indwelling catheter or immunocompromised status. The Taiwanese study looking at 84 *C. indologenes* isolates found that 98.8% of the infections were nosocomial and 79.8% of the patients had comorbidities such as cardiovascular disease (50%), diabetes (41.7%), and malignancy (25%). In that study, the case mortality rate was 25% [13].

Generally, the kidneys can enter a state of disease when they become damaged, with the most common inciting factor being diabetes. Once kidney disease, also known as renal disease, is present, the ability to effectively filter blood is impaired, leading to increased waste products and fluids in the body. In advanced or moderate to severe stages, this uremia or excess blood waste can affect other body organs. Kidney disease can most directly perpetuate heart disease and stroke. At its worst, like in the final stages of the disease or the context of renal failure, a kidney transplant is suggested.

Even though many recipients develop a urinary tract infection (UTI) after a kidney transplant, UTI after a renal transplant is still primarily an under-represented field of study. Urinary tract infections, particularly recurrent UTIs, are a common issue, affecting more than 75% of kidney transplant recipients. UTI reduces health-related quality of life and can impair graft function, potentially resulting in graft and further complications. Prospective studies on this subject are desperately needed [14].

## Conclusions

*Chryseobacterium indologenes* is a unique microbe uncommon in humans but rapidly prevalent in immunocompromised or severely ill patients. Diagnostic tests like urine and blood cultures can help identify this bacterium and provide insight into treating an infection. It is essential to appropriately identify *C. indologenes* because its varying susceptibilities to antibiotics require sensitivity testing and specific antimicrobial treatment.
